# 肺癌脑转移患者Th17细胞和IL-17水平变化的研究

**DOI:** 10.3779/j.issn.1009-3419.2013.09.07

**Published:** 2013-09-20

**Authors:** 改平 何, 彬 张, 宝文 张, 梁杰 乔, 仲兰 田, 国岩 翟, 宪伟 辛, 春 杨, 培刚 刘, 勇 张, 玲玲 徐

**Affiliations:** 300222 天津，天津市第四医院脑系科 Department of Neurology, Tianjin No.4 Hospital, Tianjin 300222, China

**Keywords:** 肺肿瘤, 脑转移, Th17细胞, IL-17, Lung neoplasms, Brain metastases, Th17 cell, IL-17

## Abstract

**背景与目的:**

Th17细胞是一种重要的辅助性T细胞，其主要分泌IL-17等细胞因子，在感染免疫、自身免疫性疾病和肿瘤免疫中均有重要意义。本研究旨在探讨Th17细胞和IL-17在肺癌脑转移患者外周血中的表达及IL-17在肺癌脑转移患者脑脊液中的表达和意义。

**方法:**

流式细胞术检测22例肺癌脑转移患者和20名正常对照外周血Th17（CD3^+^CD4^+^IL^-^23R^+^）细胞的百分率，ELISA方法检测22例肺癌脑转移患者和20名正常对照血浆IL-17水平，ELISA方法检测19例肺癌脑转移患者和16例无脑转移肺癌患者脑脊液IL-17水平。

**结果:**

肺癌脑转移患者外周血Th17细胞百分率（4.65%±0.72%）明显高于正常对照（2.71%±0.54%, *P*=0.04）；其中非小细胞肺癌（non-small cell lung cancer, NSCLC）患者和小细胞肺癌（small cell lung cancer, SCLC）患者没有差异。肺癌脑转移患者血浆IL-17水平明显高于正常对照（117.4±16.43 pg/mL和72.55±8.19 pg/mL, *P*=0.02）；其中NSCLC患者和SCLC患者没有差异。肺癌脑转移患者脑脊液IL-17水平明显高于无脑转移的肺癌患者（73.21±7.52 pg/mL和50.25±8.04 pg/mL, *P*=0.04）。

**结论:**

肺癌脑转移患者外周血Th17细胞数量增多，血浆IL-17和脑脊液IL-17水平升高，Th17细胞和IL-17可能参与了肺癌脑转移的发生和发展。

## Abstract (en)

**Background and objective:**

Th17 cells are important T helper cells, which are characterized by their production of IL-17. Th17 cells play an important role in host defense against microbial infections, autoimmune diseases and cancer. The aim of this study is to investigate the percentage of Th17 in peripheral blood lymphocyte and the level of IL-17 in serum and cerebrospinal fluid (CSF) in patients with brain metastases from lung cancer.

**Methods:**

Twenty-two patients with brain metastases from lung cancer and 20 health controls were analyzed. The percentage of Th17 cell was detected with flow cytometry using CD3^+^CD4^+^IL^-^23R^+^ marker, the level of IL-17 was measured with ELISA method.

**Results:**

The percentage of Th17 cells in patients with brain metastases from lung cancer was 4.65%±0.72%, which was remarkably higher than that in controls (2.71%±0.54%, *P*=0.04). There was no significant difference between non-small cell lung cancer (NSCLC) patients and small cell lung cancer (SCLC) patients. Serum IL-17 was remarkably increased in patients with brain metastases from lung cancer (117.4±16.43 pg/mL *vs* 72.55±8.19 pg/mL, *P*=0.02). No significant difference of the serum IL-17 was observed between NSCLC and SCLC patients. The level of IL-17 in CSF from patients with brain metastases from lung cancer was significant higher than that from lung cancer patients without brain metastases (73.21±7.52 pg/mL *vs* 50.25±8.04 pg/mL, *P*=0.04).

**Conclusion:**

Th17 cells and IL-17 increase in patients with brain metastases from lung cancer. It may involve in the pathogenesis of brain metastases from lung cancer.

肺癌是导致患者死亡数量第一的恶性肿瘤^[1]^。美国2012年的数据显示，肺癌新发病例约为22.6万，其中男性11.6万，女性11.0万；而16.0万例患者死于肺癌，其中男性8.8万，女性7.2万。我国肿瘤登记资料显示，肺癌已经位列城市肿瘤发病率和死亡率的第一位^[2]^。脑部是肺癌远处转移最常见的部位之一，肺癌脑转移的发生率为23%-65%，是脑转移肿瘤中最常见的类型。随着肺癌发病率的逐年上升，头颅CT、磁共振和PET-CT等各种诊疗技术的提高，肺癌脑转移的检出率也随之增加。肺癌脑转移预后较差，其中位生存期为3.1个-12个月^[3]^。

Th17细胞是一种新发现的辅助性T细胞。Th0细胞在IL-23等的刺激下分化成为Th17细胞，其主要分泌IL-17等细胞因子，视黄醇相关孤儿受体γt（retinoid-related orphan receptor-gamma t, RORγt）是其重要的转录因子。目前研究^[4]^发现Th17细胞在感染免疫、自身免疫性疾病和肿瘤免疫中均居于重要意义。我们对22例伴有脑转移的肺癌患者外周血Th17细胞、血浆和脑脊液IL-17水平进行了研究，现报道如下。

## 1 资料和方法

### 1.1 病例资料

收集我院2010年1月-2012年12月就诊肺癌脑转移患者22例，其中男13例，女9例，中位年龄59（范围34-78）岁。所有病例均经病理学或者组织学确诊，其中小细胞肺癌（small cell lung cancer, SCLC）12例，非小细胞肺癌（non-small cell lung cancer, NSCLC）10例（其中腺癌7例，鳞癌3例）。根据UICC分期，所以患者均为Ⅳ期。20名健康对照来自我院查体中心，其中男10名，女10名，中位年龄57（范围40-75）岁。肺癌脑转移组和健康对照组在年龄和性别方面无明显差异。研究方案已经天津市第四医院伦理委员会批准，所有研究对象均已签署知情同意书。

### 1.2 仪器

鼠抗人PE-IL23R、鼠抗人FITC-CD4、鼠抗人APC-CD3、鼠抗人PE-IgG1鼠抗人FITC-IgG1、鼠抗人APC-IgG1、溶血素均为美国BD PharMingen公司产品；IL-17酶联免疫吸附试验（ELISA）检测试剂盒为美国R & D公司产品；流式细胞仪为美国BD公司的BD FACSCalibur；酶标仪为美国BioTek公司Elx800光吸收酶标仪。

### 1.3 流式细胞检测

采集患者及正常对照空腹外周静脉血2 mL，肝素抗凝。每流式管中分别加入全血100 μL。各管中分别加入APC-CD3、FITC-CD4、PE-IL23R及同型IgG1对照单克隆抗体20 μL，混匀，室温避光孵育30 min。加入溶血素2 mL，室温避光10 min溶解红细胞。1, 500 rpm，离心5 min。去上清。加入PBS 5 mL，1, 500 rpm，离心5 min，去上清。重复2次。200 μL多聚甲醛固定20 min。

流式细胞仪检测分析。应用IgG1作为同型对照，去除背景荧光。在FACSCalibur流式细胞仪上使用CellQuest3.0软件（Macintosh）获取细胞并进行分析。因为外周血DC细胞前向角（FSC）与单核细胞相似，侧向角（SSC）与淋巴细胞相似，所以应用FSC和SSC进行设门，选定单个核细胞群（包括淋巴细胞和单核细胞)，去除粒细胞和细胞碎片。获取20, 000个细胞进行分析。

### 1.4 脑脊液和血浆的采集和保存

采集无腰穿禁忌症的患者脑脊液2 mL，-20 ℃冻存备用。采集患者及正常对照空腹外周静脉血2 mL，肝素抗凝。1, 500 rpm，离心5 min。取上清，-20 ℃冻存备用。

### 1.5 ELISA检测

操作按照IL-17 ELISA试剂盒说明书。具体如下：加入标准品/标本稀释液1.0 mL至冻干标准品中，待彻底溶解后，静置15 min，混匀。稀释为以下浓度：2, 000 pg/mL、1, 000 pg/mL、500 pg/mL、250 pg/mL、125 pg/mL、62.5 pg/mL、31.25 pg/mL、0 pg/mL。稀释浓缩生物素化抗体（1:100）和浓缩酶结合物（1:100）。标本激活：将450 μL标准品/标本稀释液加入10 μL血浆标本。加20 μL HCl(1 mol/L)，盖紧，上下混匀。4 ℃放置60 min。加入20 μL NaOH（1 mol/L），盖紧，上下混匀。除空白孔外，分别将标本或不同浓度品（100 μL/孔）加入相应孔中；用封板胶纸封住反应孔，37 ℃孵育60 min。洗板4次。除空白孔外，加入生物素化抗体工作液（100 μL/孔）；用封板胶纸封住反应孔，37 ℃孵育60 min。洗板4次。除空白孔外，加入酶结合物工作液（100 μL/孔）；用封板胶纸封住反应孔，37 ℃孵育30 min。洗板4次。加入显色剂（100 μL/孔），避光37 ℃显色20 min。加入终止液（100 μL/孔），混匀。立即应用酶标仪测定OD_450_值。

### 1.6 统计学方法

统计分析采用SPSS 11.0软件完成。计量资料用Mean±SD表示，组间比较应用方差分析。*P* < 0.05为差异具有统计学意义。

## 2 结果

### 2.1 肺癌脑转移患者外周血Th17细胞比例高于正常对照

我们应用流式细胞术对肺癌脑转移患者（*n*=22）外周血Th17细胞（CD3^+^CD4^+^IL^-^23R^+^细胞）进行检测，发现其数量较正常对照（*n*=20）明显增多（分别为4.65%±0.72%和2.71%±0.54%，*P*=0.04）。脑转移的NSCLC患者（*n*=10）外周血Th17细胞比例为4.97%±1.14%，而脑转移的SCLC患者（*n*=12）为4.38%±0.95%，两者没有明显差异（*P*=0.69）（图 1）。

**1:**
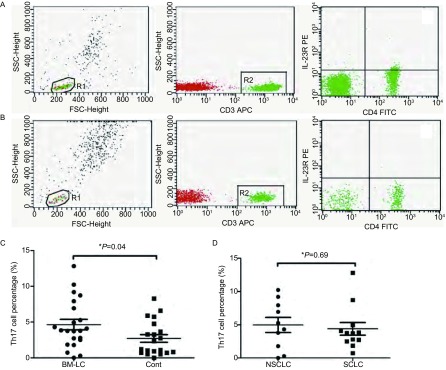
肺癌脑转移患者和正常对照外周血Th17细胞检测。A：肺癌脑转移患者外周血Th17流式检测图；B：正常对照外周血Th17流式检测图；C：肺癌脑转移患者外周血Th17细胞比例高于正常对照组；D：NSCLC和SCLC脑转移患者外周血Th17细胞比例没有明显差异。 Peripheral blood Th17 cells in patients with brain metastases from lung cancer and health controls. A: Th17 cell was detected with flow cytometry using CD3^+^CD4^+^IL^-^23R^+^ marker in patients with brain metastases from lung cancer; B: Th17 cell was detected with flow cytometry using CD3^+^CD4^+^IL^-^23R^+^ marker in health controls; C: The percentage of Th17 cells in patients with brain metastases from lung cancer was higher than that in health controls; D: The percentage of Th17 cells in patients with NSCLC and SCLC. BM-LC: brain metastases from lung cancer; Cont: health controls. SCLC: small cell lung cancer; NSCLC: non-small cell lung cancer.

### 2.2 肺癌脑转移患者血清IL-17水平高于正常对照

我们应用ELISA方法检测22例肺癌脑转移患者血清IL-17水平，并与正常对照（*n*=20）的血清中IL-17进行比较。结果显示，伴有脑转移的肺癌患者血清IL-17水平为（117.4±16.43）pg/mL，正常对照血清IL-17水平为（72.55±8.19）pg/mL，两者间差异具有统计学意义（*P*=0.02）。脑转移的NSCLC患者（*n*=10）血清IL-17为104.4%±19.83%，而脑转移的SCLC患者（*n*=12）为128.3%±25.56%，两者没有明显差异（*P*=0.48）（图 2）。

**2:**
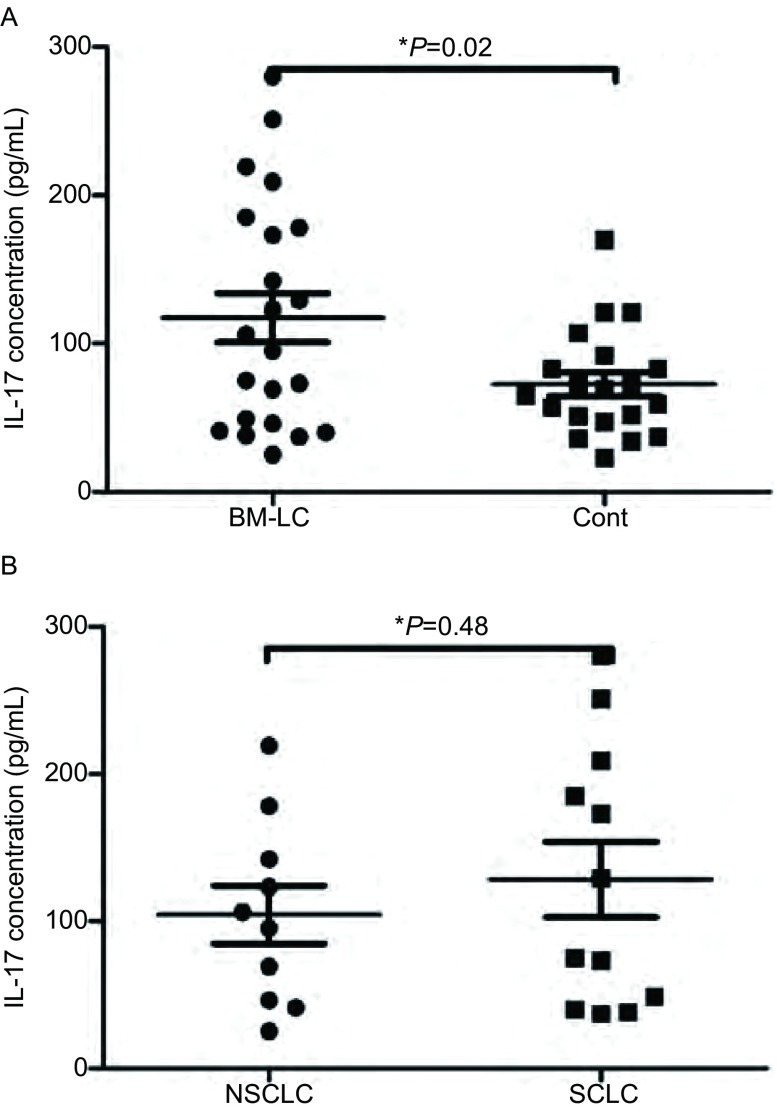
肺癌脑转移患者和正常对照血浆IL-17检测。A：肺癌脑转移患者血清IL-17浓度高于对照组；B：NSCLC和SCLC脑转移患者血清IL-17浓度。 Serum IL-17 concentration in patients with brain metastases from lung cancer and health controls. A: Serum IL-17 concentration in patients with brain metastases from lung cancer was higher than that in health controls; B: Serum IL-17 concentration in patients with NSCLC and SCLC. BM-LC: brain metastases from lung cancer; Cont: health controls.

### 2.3 肺癌脑转移患者脑脊液IL-17水平高于无脑转移的肺癌患者

我们应用ELISA方法检测19例肺癌脑转移患者脑脊液中IL-17水平，并与没有脑转移的肺癌患者（*n*=16）的脑脊液中IL-17进行比较。结果显示，伴有脑转移的肺癌患者脑脊液IL-17水平为（73.21±7.52）pg/mL，没有脑转移的肺癌患者脑脊液IL-17水平为（50.25±8.04）pg/mL，两者间差异具有统计学意义（*P*=0.04）（图 3）。

**3:**
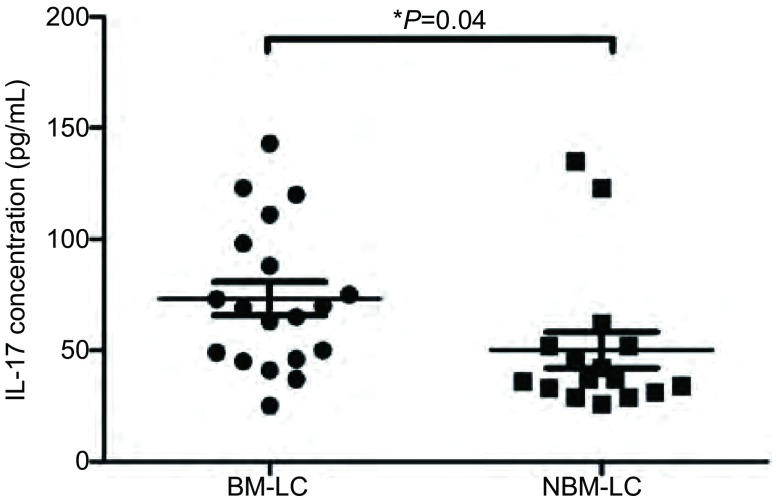
肺癌脑转移患者和无脑转移的肺癌患者脑脊液IL-17浓度。BM-LC：肺癌脑转移；NBM-LC：无脑转移的肺癌患者。 Cerebrospinal fluid IL-17 concentration in patients with brain metastases from lung cancer and lung cancer without brain metastases. BM-LC: brain metastases from lung cancer; NBM-LC: lung cancer without brain metastases.

## 3 讨论

Th17是一种新发现的能够分泌IL-17的T细胞亚群，在自身免疫性疾病和机体防御反应中具有重要的意义。β转化生长因子（transtroming growth factor β, TGF-β）、IL-6、IL-23、IL-21和IL-1在Th17细胞的分化形成过程中起着积极的促进作用，而γ干扰素（interferon gamma, IFN-γ）、IL-4、细胞因子信号传送阻抑蛋白3（Socs3）和IL-2则抑制它的分化。Th17是由TH0细胞在IL-6和IL-23的刺激下分化而成的辅助性T细胞，主要分泌IL-17、IL-22等促炎症因子，RORγt是其最重要的转录因子，其他重要的转录因子还包括信号转导和转录激活因子3（Stat3）、核受体ROR-α、IFN调节因子4（IRF-4）、B细胞激活转录因子（B-ATF）和低氧诱导因子1α（HIF1-α）^[4]^。

Th17细胞在肿瘤发病和进展中的作用目前还不清楚。目前研究发现，很多肿瘤患者存在Th17细胞浸润，包括淋巴瘤、骨髓瘤、乳腺癌、大肠癌、胃癌、肝细胞肝癌、黑色素瘤、卵巢癌、胰腺癌和前列腺癌等。肿瘤浸润的Th17细胞除了分泌细胞因子IL-17外，还分泌大量IL-8、TNF-α、IL-6、IL-10和TGF-β1，通过这些细胞因子，在肿瘤微环境中发挥作用。虽然现在研究已经明确肿瘤微环境中存在大量Th17细胞，但是对于它们在肿瘤中的作用还有争议^[5]^。

多数研究显示Th17细胞促进肿瘤生长。肿瘤小鼠模型和肿瘤患者的临床研究均发现IL-17和Th17细胞具有促进肿瘤生长的特性。IL-17和Th17细胞促进肿瘤生长的机制包括参与新生血管的形成和分泌细胞因子改变肿瘤微环境。在裸鼠内，IL-17通过升高IL-6和IL-8水平，招募巨噬细胞到肿瘤部位，促进人类宫颈癌的生长^[6]^。结肠腺癌小鼠模型的研究显示，IL-17诱导肿瘤新生血管形成，诱导肿瘤细胞和纤维母细胞分泌VEGF、PGE2、角质细胞来源趋化因子和一氧化氮等血管形成因子^[7]^。非小细胞肺癌研究显示，IL-17通过CXCR2依赖的新生血管形成方式，诱导血管形成。人类肝细胞肝癌研究显示，肿瘤浸润的IL-17细胞促进新生血管形成，促进肿瘤生长。IL-17诱导IL-6产生，IL-6激活肿瘤信号Stat3，上调肿瘤生存和血管形成基因^[8]^。

最近几项临床试验也证实了IL-17和Th17在不同肿瘤中具有促进肿瘤生长的作用。Hahn等^[9]^报道Th17细胞随着胃十二指肠部位癌前病变的进展而数量增加。Tosolini等^[10]^分析了125例大肠癌患者的冰冻肿瘤标本，发现Th17高表达的患者预后差，而Th1高表达的患者预后好。Wu等^[11]^研究发现，急性髓系白血病患者外周血Th17细胞数量较正常对照明显升高，IL-17的水平也明显升高。多发性骨髓瘤患者Th17细胞和IL-17水平升高，并抑制效应免疫功能^[12]^。卵巢癌患者IL-17水平升高，招募TNF-α，促进肿瘤生长^[13]^。

虽然大多数研究显示Th17细胞促进肿瘤生长，但也有研究显示Th17细胞具有抑制肿瘤生长效应。肿瘤小鼠模型显示Th17细胞能直接根除肿瘤细胞。Muranski等^[14]^研究显示肿瘤特异性Th17细胞能使小鼠模型中的黑色素瘤消退，这种治疗作用依赖于产生的IFN-γ。主动转染分泌IL-17的CD8 T细胞能增强抗肿瘤免疫，使巨大的B16黑色素瘤消退。转染分泌IL-17的CD8 T细胞能转化成产生IFNγ的效应T细胞，发挥抗肿瘤效应。除了直接根除肿瘤细胞效应外，Th17细胞还具有其他间接抗肿瘤效应，如招募其他肿瘤特异性免疫细胞或者增强它们的抗肿瘤效应。Benchetrit等^[15]^研究显示，转染IL-17到裸鼠中，IL-17不能抑制造血肿瘤的生长；而转染到具有免疫功能的小鼠中，IL-17能抑制造血肿瘤的生长，这种抗肿瘤效应是通过增加肿瘤特异性细胞毒性T淋巴细胞实现的。Th17细胞通过促进肿瘤特异性CD8 T细胞激活方式，发挥间接抗肿瘤效应。应用肿瘤特异性Th17细胞进行主动T细胞治疗，能招募树突细胞进入肿瘤部位和附近的淋巴结，启动强大的抗肿瘤CD8 T细胞反应。IL-17缺陷小鼠肿瘤及其附近淋巴结中IFN-γ NK细胞和肿瘤特异性IFN-γ T细胞数量减少，导致肿瘤生长加速，并导致肿瘤转移。IL-17对于肿瘤的影响取决于已经存在的主动免疫系统。存在淋巴细胞的情况下，IL-17抑制肿瘤生长；没有淋巴细胞时，IL-17促进肿瘤生长和转移。绝大部分支持Th17细胞具有抗肿瘤效应的证据都来自于小鼠模型，所以，Th17细胞是否在人体内有抗肿瘤作用还不清楚。

我们研究结果显示，肺癌脑转移患者外周血Th17细胞数量和血浆IL-17的水平均高于正常对照，说明Th17细胞和IL-17在肺癌中属于预后不良指标，此类患者发生脑转移的几率较大。Th17细胞数量和IL-17的水平与肺癌的病理类型没有明显的相关性，不具有肿瘤类型的特异性。脑转移肺癌患者的脑脊液中IL-17表达水平高于没有脑转移的肺癌患者，提示Th17细胞和IL-17参与了肺癌发生脑转移的机制。

与其他肿瘤研究一样，Th17细胞和IL-17在肺癌中的作用是促进肿瘤生长还是抑制肿瘤生长还没有定论。早期的研究^[16]^显示肿瘤来源的Th17细胞和IL-17是肺癌以后良好的标记，局限SCLC患者Th17细胞数量高于广泛转移的SCLC患者。Th17细胞能诱导肺癌CD8^+^细胞毒性T淋巴细胞激活，发挥抗肿瘤效应^[17]^。但我国近期的临床研究^[18, 19]^发现，Th17和IL-17在肺癌患者外周中均升高，并且随着肿瘤的进展，数量逐渐升高。手术切除肺癌后患者Th17细胞和IL-17水平明显下降。提示Th17细胞和IL-17是肺癌患者预后不良的指标。我们的研究结果也显示，Th17细胞和IL-17参与了肺癌脑转移，属于预后不良的指标。

综上所述，Th17细胞和IL-17可能参与了肺癌脑转移的发生，对其进行深入研究有助于揭示Th17细胞在肺癌发生和转移的机制，对于肺癌的诊断和免疫治疗具有重要意义。
